# Antimalarial Properties of *Sida cordifolia* L. Leaf Extract in Mice: Survivability Depends Less on Parasitaemia Suppression

**DOI:** 10.1155/2023/5560711

**Published:** 2023-12-23

**Authors:** Samuel Ansong, Desmond Nkrumah, Reinhard Isaac Nketia, Samuel Oppong Bekoe, Abena Amponsaa Brobbey, Evelyn Asante-Kwatia, Gustav Komlaga

**Affiliations:** ^1^Department of Pharmacognosy, KNUST, Kumasi, Ghana; ^2^Department of Pharmaceutical Chemistry, KNUST, Kumasi, Ghana

## Abstract

*Sida cordifolia* has been used to treat malaria in Ghana albeit without scientific evidence of antimalarial activity and safety. This work aimed to assess the antimalarial properties and acute toxicity of the aqueous leaf extract of *S. cordifolia* in murine models. Aqueous extract of the plant was analysed for both suppressive and curative antimalarial properties in chloroquine-sensitive ANKA strains of rodent *Plasmodium berghei*-infected mice. Acute toxicity evaluation was performed in rats according to the OECD 425 guidelines. The extract displayed antiplasmodial activity *in vivo* with ED_50_ of 117.49 ± 15.22 mg/kg and 144.84 ± 18.17 mg/kg in suppressive and curative studies, respectively. The highest % parasitaemia suppression exerted was 76.90 ± 0.64% and 61.50 ± 0.97%, respectively, in the suppressive and curative studies. Survival of infected mice treated with the extract was significantly prolonged. This was dependent on the dose of the extract but imperfectly related to the % parasitaemia suppression. Related antimalarial parameters including percentage hematocrit, changes in body weight, and temperature of experimental mice indicated alleviation of malarial symptoms of treated animals. The extract did not show toxicity in rats. *Sida cordifolia* L. has antimalarial properties, and was safe. It suppressed parasitaemia in both suppressive and curative studies, was not toxic to animals and prolonged the life of infected animals under treatment. This, therefore, justifies the traditional use of *S. cordifolia* for the treatment of malaria in Ghana.

## 1. Introduction

There is more access to antimalarial drugs in Ghana and Africa currently than it was some decades past. This notwithstanding, many Ghanaians, and by extension, Africans including urban dwellers continue to use herbal products or medicinal plants for the treatment of malaria and other diseases. Various ethnobotanical surveys [1–3] have inventoried many medicinal plants used in traditional medicine to treat malaria. Meanwhile, malaria remains a devastating health problem affecting the countries of sub-Saharan Africa the most. Worse of it, earlier gains made in the control of the disease had been disrupted as a result of the Coronavirus disease 2019 (COVID-19) pandemic [4].

In 2019, before the pandemic struck, the number of deaths stood at 568 000 and this increased to 625 000 in the first year of the pandemic. Though it reversed to an estimated 619,000 malaria deaths globally in 2021, malaria cases on the other hand continued to increase globally reaching 247 million in 2021 as compared to 245 million in 2020 and the cases were lower in earlier years [5].

According to the World Health Organization (WHO), about 95% of malaria cases and 96% of the global deaths occurred in the WHO African Region. Approximately 80% of all malaria deaths in the African Region occurred among children under the age of five [5]. Various treatment strategies including combination therapies have been used over the years in the control of the disease. A more recent addition was the use of a malaria vaccine among children under five [5]. Yet, the use of medicinal plants in sub-Saharan Africa remains substantial.

One such plant is *Sida cordifolia* (Malvaceae). *S. cordifolia* is a perennial short, erect, and pubescent woody shrub that grows up to 0.75–1.5 meters in height. Its roots and stem are stout and strong [6]. It is thus called “obraneatuata” in the Akan language of Ghana. The leaves, at maturity, are simple and serrate; alternate, orbicular, ovate, ovate-oblong or cordate, margin crenate, base cordate, petiolated, stipulate, and stipules linear. The leaves are 2.5–5 cm long and 1.8–3 cm broad.

Ethnobotanically, poultices are prepared from the crushed leaves and used as local anesthetics [7]. The aqueous extract is traditionally used in treating headaches, aching joints and bones, oedema, cough, gastrointestinal and urinary infections, debility, skin ailments, weight loss, rheumatism, stomatitis, stomach upset, diarrhoea, premature ejaculation, miscarriage, malaria, fever, bronchitis, variola, and sciatica [8]. In Ghana and Cameroon, *S*. *Cordifolia* is reported to relieve the attendant pain of pregnancy and labour [9].

Reported biological activities include analgesic, anti-inflammatory [8], antipyretic, antiulcer [10], and antioxidant [11] activities. The aqueous extract of the whole plant at a dose of 400 mg/kg has been reported to significantly reduce blood glucose levels as well as total cholesterol, triglycerides, low-density lipoprotein, plasma creatinine, and plasma-urea nitrogen levels [12]. Vasicine, an alkaloid isolated from the leaves of *S. Cordifolia*, has also been reported to produce hypotension and bradycardia by direct and indirect stimulation of muscarinic receptors and by reduction of peripheral resistance [13].

As part of a series of evaluations of medicinal plants used to treat malaria in Ghana, this project sought to assess the antimalarial properties and toxicity of the aqueous extract of the *S*. *cordifolia* L. prepared according to traditional methods. *S. cordifolia* was among the medicinal plants cited by herbalists for treating malaria in Ghana with an appreciable proportion of respondents having knowledge about it [3]. However, no scientific data exist to support its antimalarial activity and safety, although a related genus *Sida acuta* was reported to have antiplasmodial activity [14]. Data from this study would, therefore, validate or disprove the traditional antimalarial claims about the plant. The study would provide an avenue for further study that could involve the isolation and characterization of the antimalarial compounds of different chemical structures capable of being effective against antimalarial drug-resistant strains of *P. falciparum*.

## 2. Materials and Methods

### 2.1. Plant Material Collection

The leaves of *S*. *cordifolia* were harvested in March 2021 at Kwahu Asakraka (N6°40′27.714″ and W1°33′58.7412″) in the Eastern Region of Ghana. The plant material was authenticated by Dr George H. Sam of the Department of Herbal Medicine, and a herbarium specimen (KNUST/HM1/2015/L002) was deposited at the Herbarium of the Department of Herbal Medicine, Faculty of Pharmacy and Pharmaceutical Sciences, Kwame Nkrumah University of Science and Technology (KNUST), Kumasi, Ghana.

### 2.2. Plant Material Processing

Debris and foreign matter were manually removed from the plant material. It was washed under running water, cut into smaller sizes, and dried under shade for 14 days. The dried materials were then milled into coarse powder using a mechanical grinder and stored until required for use.

### 2.3. Extraction of Plant Material

The plant material was extracted according to the traditional approach except for the specified quantity. About 106.95 g of the powdered material was boiled in 3 L of water on an electric stove (Professional 2 Burner Electric Hot plate, China) for 30 min. The boiled mixture was filtered hot, first with a piece of cotton wool, followed by Whatman filter paper no. 1 (Whatman®, England). The filtrate was freeze-dried (Thermo Scientific Heto PowerDry LL3000), and the dried extract (15.146 g) was labelled and stored in a desiccator until needed for use.

### 2.4. Phytochemical Screening

The powdered material was subjected to basic qualitative phytochemical screening for phytochemical constituents using standard procedures [15].

### 2.5. Experimental Animals

Three (3) healthy nulliparous and nonpregnant female Swiss albino rats of both sexes (200–205 g and 8–10 weeks of age) were used for the acute toxicity study, while forty (40) healthy adult Swiss albino mice of both sexes (20–28 g and 6–8 weeks of age) were used for the antimalarial investigations. Animals were obtained from the Noguchi Memorial Institute for Medical Research (NMIMR), University of Ghana, Accra, Ghana. They were housed at the animal house of the Department of Pharmacology, KNUST, in aluminum cages with softwood shavings and chips as beddings. Animals were exposed to a 12/12 dark-light cycle and had free access to a pellet diet and clean drinking water. Animals were acclimatized for 1 week before the beginning of the experiment. Ethical approval for the study was granted by the KNUST Animal Research Ethics Committee (certificate no. KNUST 001). All other institutional standard operating procedure for animal research was duly observed throughout the entire duration of the study. At the end of the experiment, surviving animals were humanely euthanized by manually applying blunt force trauma to the head by skilled personnel [16, 17] at the animal house. This was performed away from other animals to save them from hearing the distress vocalizations or sensing distress pheromones. Death was confirmed by physical observation for the absence of pulse, breathing, corneal reflex, and response to firm toe pinch. All euthanized and nonsurviving animals were buried in a designated location within our Physic Garden.

### 2.6. Acute Toxicity Study

The OECD guidelines 425 [18] for the conduct of acute toxicity study was followed. The limit test at the single dose of 2000 mg/kg body weight was used as no data on demonstrable toxicity of *S. cordifolia* exists. Rats were fasted overnight but were allowed unlimited access to water, prior to the experiment. Animals were weighed and administered equivalent volumes of the aqueous solution of the crude extract by oral gavage. Food was withheld for a further three hours, during which the rats were separately observed at 30 min intervals for the first 1 h and then occasionally for the next 24 h with special attention given in the first 4 h. Thereafter, observation was made daily for the next 13 days for manifestations of signs of toxicity, including gross physical and behavioural changes such as rigidity, sleepiness, diarrhoea, abnormal secretion or hair erection, and/or death.

### 2.7. Parasite Preparation

Blood-stage samples of chloroquine-sensitive ANKA strains of the rodent *Plasmodium berghei*, kept in liquid nitrogen at the Central Laboratory of KNUST, were used in the antiplasmodial experiment. Parasites were passaged in Swiss albino mice as doners. The parasitized, donor mice (with about 25% parasitaemia) were euthanized with diethyl ether, followed by a collection of blood samples by cardiac puncture into heparinised vacutainer tubes. The infected blood (parasitaemia 30%) was diluted with physiological saline (0.9%) such that 1 mL of blood contained 1 × 10^7^ parasitized erythrocytes [19]. An aliquot of 0.2 mL corresponding to 2 × 10^6^ infected red blood cells (RBCs) of this suspension was administered intraperitoneally (i. p.) to the mice for the antiplasmodial assay. All surviving infected experimental mice were euthanized at the end of the experiment on day 30.

### 2.8. Peter's Four-Day Suppressive Test

Antimalarial activity of the test extracts was performed in a 4-day suppressive standard test [20] using mice inoculated with parasites. The inoculated mice were randomly divided into 6 groups of 4. These groups were treated separately with 50, 100, 200, and 400 mg/kg/day of extract via oral gavage (p.o.) three hours following inoculation. Artesunate at the dose of 4 mg/kg/day and an equivalent volume of normal saline were also given to the positive and negative control groups, respectively. Treatment was carried out for four consecutive days. On the fifth day (i.e., 24 h after the last dose or 96 h postinfection), thin blood smears were prepared from the tail vein of each mouse on slides, fixed with absolute methanol, and stained with 10% Giemsa stain for 15 min. Parasitaemia was then determined in each case by counting the number of both parasitized and nonparasitized erythrocytes in five randomly selected fields under a magnification of an ×100 objective lens of a light microscope (Leica DM750 HD microscope, Switzerland).

The drug concentrations at which parasitaemia was reduced by 50% (ED_50_) and 90% (ED_90_), were, respectively, determined. Moreover, the degrees of parasitaemia suppression (% parasitaemia suppression) for each treatment were determined. Changes in body weight and rectal temperature were, respectively, measured by using a digital weighing balance (Sartorius, Hamburg, Germany) and a rectal digital thermometer for animals [21]. Packed cell volume or hematocrit was determined by analysing the blood samples of experimental mice, collected in heparinised ethylenediaminetetraacetic acid (EDTA) tubes. The blood was analysed with an automated haematological analyser (Sysmex XN-550 haematological analyser). Animal mortalities by the 30^th^day after infection were monitored. Deaths, if any, were recorded during the period, and the median survival time was estimated from the survival curves for each group.

Parasitaemia was calculated by using the following formula:

(1) $$\begin{array}{c} \text{parasitaemia}=\frac{\text{number of parasitized RBC}}{\text{total number of RBC counted}}\times 100. \end{array}$$

The % parasitaemia suppression was calculated as follows:

(2) $$\begin{array}{c} \frac{A-B}{A}\times 100, \end{array}$$

where *A* is the parasitaemia in the negative control group and *B* is the parasitaemia in the test group.

### 2.9. Rane's Curative Test

The curative antimalarial activity of the crude extract was assessed following the methods of Komlaga et al. [22]. Mice infected 3 days (72 h) earlier were administered the extract at 50, 100, 200, and 400 mg/kg p.o, and thence every 24 h for 3 consecutive days. Artesunate was used as the reference drug at 4 mg/kg/day p.o. The negative control group received 0.2 mL of normal saline. Parasitaemia was determined on the day of the first treatment (3 days postinfection) and 24 hours following the last administration of the extract (7 days postinfection). ED_50_, ED_90_, and % parasitaemia suppression were subsequently estimated. In addition, changes in body weight and rectal temperature of mice between days three and seven were determined. Survival was monitored until the 30^th^ day of the infection, the survival curve was drawn, and the median survival time was estimated.

## 3. Data Analysis

Data were analysed using GraphPad Prism (GraphPad Software, version 8.0.2, San Diego, CA, USA) and presented as mean ± SEM. Comparisons were made between the negative control group and treatment groups including the positive control group by using the one-way analysis of variance (ANOVA) followed by Dunnet's multiple comparison test. Comparisons within test groups were also made by using two-way ANOVA followed by Turkey's multiple comparison test. Changes in rectal temperature and body weight were compared using two-way ANOVA followed by Dunnet's multiple comparison test. Survival curves were compared under the log-rank (Mantel–Cox) test, and the differences were compared at a *p* value < 0.0001.

## 4. Results

### 4.1. Basic Qualitative Phytochemical Screening

The powdered plant material tested positive for tannins, saponins, reducing sugars, triterpenoids, and coumarins, while it tested negative for flavonoids, alkaloids, and phytosterols.

### 4.2. Acute Oral Toxicity Assessment

No gross physical and behavioural changes such as rigidity, sleepiness, diarrhoea, abnormal secretion, or hair erection for 24 h were observed during the acute toxicity study of the extract in rats. All rats survived the two-week observation period at the dose level of 2000 mg/kg body weight.

### 4.3. Antimalarial Activity

**Figure 1:**
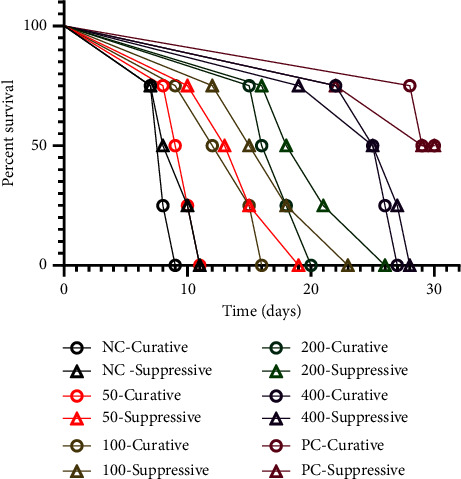
Survivability graph for both curative and suppressive studies.

The suppressive activity of the extract and the median survival time of infected mice both showed dose-dependency for both suppressive and curative studies (Table 1 and Figure 1). However, the percentage parasitaemia suppression and the median survival time were both higher in the suppressive study than in the corresponding curative study. The percentage parasitaemia suppression of the extract showed an imperfect correlation (Pearson's *r* is 0.921) with the median survival time of extract-treated infected mice. Generally, mice showed significantly greater survivability (*P* value < 0.0001) in the suppressive study than in the curative group.

**Figure 2:**
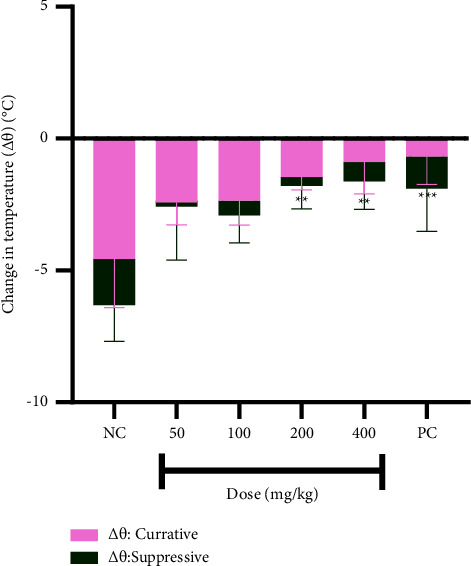
Changes in body temperature (Δ*θ*) of mice in both studies. Data are presented as mean ± SEM, *n* = 4, NC: vehicle-treated group, and PC: artesunate (2 mg/kg). Values are statistically significant at ^*∗∗∗*^*p* < 0.001 and ^*∗∗*^*p* < 0.05 in comparison with the negative control of the curative study.

The infection induced a reduction in the body temperature, which was upturned in treated mice. The reduction was generally higher in the curative study than in the suppressive study. The decrease in temperature showed dose-dependency in the curative study, while that of the suppressive study did not relate to the dose of the extract (Figure 2).

**Figure 3:**
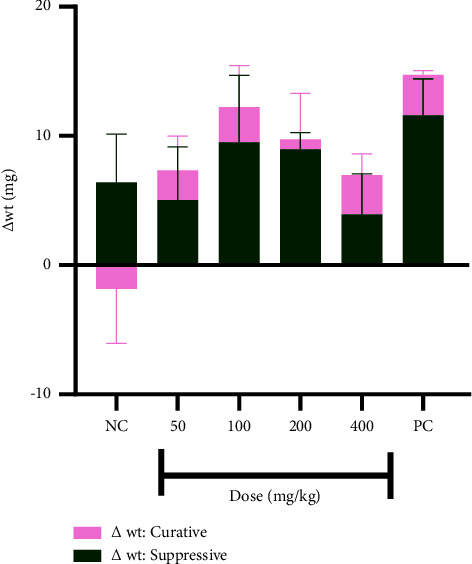
Changes in body weight of mice in both studies. Data are presented as mean ± SEM, *n* = 4, NC: vehicle-treated group, and PC: artesunate (2 mg/kg).

The extract showed no effect on the body weight of mice, neither did the parasite infection, in the suppressive study (Figure 3). It, however, reversed the loss of body weight, due to the infection, in the curative studies.

**Figure 4:**
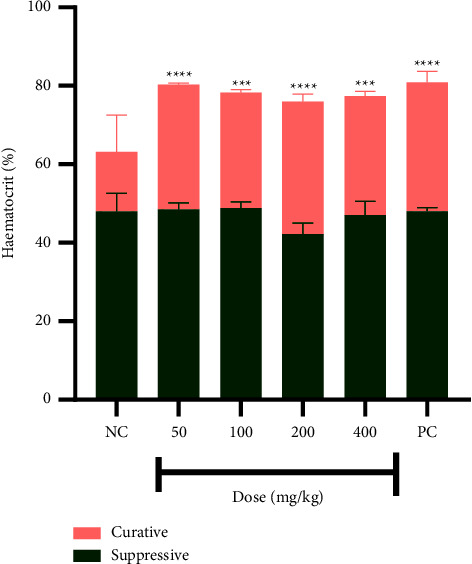
Percentage of hematocrit of mice in both studies. Data are presented as mean ± SEM, *n* = 4, NC: vehicle-treated group, and PC: artesunate (2 mg/kg). Values are statistically significant at ^*∗∗∗∗*^*p* < 0.0001 and ^*∗∗∗*^*p* < 0.001 when compared with the negative control of the curative study.

There is a higher, treatment-independent level of hematocrit (HGT) in the suppressive study. There was generally reduced, dose-independent level of HCT in the curative study (Figure 4).

## 5. Discussion

Aqueous decoction, by boiling the leaves of *S. Cordifolia,* is used in the traditional treatment of malaria. In order to validate this traditional use, the study attempted to mimic the traditional extraction approach, by boiling the powdered plant material in water for 30 minutes, to obtain the aqueous extract. The extract showed parasitaemia suppression in both the suppressive and curative studies with ED_50_ of 117.49 mg/kg in the suppressive study and 144.84 mg/kg in the curative study. This suggests the efficacy of the extract in treating unestablished/fresh infections. However, neither the extract nor artesunate produced 100% parasitaemia suppression. At the highest dose of 400 mg/kg of extract, parasitaemia was suppressed by 76.90% in the suppressive study and by 61.50% in the curative study. The positive control (artesunate) suppressed parasitaemia by 95.12% in the suppressive assay and by 92.44% in the curative assay. On the other hand, untreated mice (normal control) experienced no parasite suppression (Table 1) as the parasites were not exposed to any drug.

The enhanced antimalarial effect (low ED_50_ value of extracts and high % of parasitaemia suppression) observed in the suppressive study was probably due to low parasite load during treatment as compared to high parasite load in the curative study. The high parasite load was due to the inoculated parasites undergoing multiple life cycles during the 72 h before treatment. On the other hand, parasites in the suppressive study did not undergo any multiplication before treatment began and hence the parasite load at treatment was the same number of parasites inoculated into the mice as the mice were treated 3 h after parasite infection. Convergence of antimalarial activity at the generic level has been noticed for *S*. *acuta*, which showed *in vitro* activity against clinical isolates of *P. falciparum* [14]. Cryptolepine, a known antimalarial alkaloid first obtained from *Cryptolepis sanguinolenta*, had been cited in the Sida genus [23], and this could largely be responsible for the antimalarial property of the plant.

The survival curve illustrated by the Kaplan–Meier plot shows the survival of the mice following parasite inoculation (Figure 1). From the curve, the median survival time, which was the time taken for half-infested mice to remain alive in each group, was estimated. This was significantly prolonged (*P* value < 0.0001) for mice treated with the extract, except at 50 mg/kg dose, in both studies (Table 1). This suggests the extract's ability and usefulness to prolong the survival of infected animals at doses of 100 mg/kg and above.

The median survival times for the two treatments were statistically comparable, even though their respective % parasitaemia suppressions (Table 1) were significantly different (*P* < 0.0001).

A look at both the experiments showed the superior effect of the dose on the survivability of infected mice compared to the % parasitaemia suppression. In a multiple variable analysis, the median survival time did not show a perfect correlation with the % parasitaemia suppression (Pearson correlation coefficient, *r* = 0.921). However, it was instead dose-dependent. This reveals the significant impact of the dose of the extract, instead of the percentage parasitaemia suppression, on the survival time of treated mice. It was therefore assumed that properties, other than the antiplasmodial property of the phytoconstituents, might be largely responsible for the extended survival observed in the extract-treated mice. These bioproperties may include central nervous system activation by ephedrine, a powerful stimulant, identified in the *S*. genus [24]. The activation speeds up communication between the brain and body, increasing heart rate, blood pressure, and body temperature [25]. By increasing brain activity, the ephedrine increased alertness and physical activity but reduced tiredness. This could partly account for the extended survival time observed in the extract-treated mice. Other activities may include analgesic, anti-inflammatory, antipyretic, and antioxidant activities which were earlier reported for the plant [8, 10, 11]. Various phytochemical classes present in the plant material have been cited to be responsible for some of these bioproperties. For example, alkaloids, terpenoids, tannins, saponins, phytosterols, and flavonoids have variously been implicated for anti-inflammatory activity [26–37]. Also, alkaloids, terpenoids, tannins, saponins, phytosterols, and flavonoids have been implicated for antioxidant activity [36, 38–47]. On the other hand, the long median survival time of 29.5 days enjoyed by the mice treated with PC (artesunate) in both the studies could be due to near eradication of the parasite; the PC-treated mice had a percentage parasitaemia suppression of more than 90% in both studies. These observations, therefore, suggest the potential benefit of plants, which, although may show weak antiplasmodial activity, prove useful in the management of malaria. Our observations may not be peculiar to only medicinal plants used in the traditional management of malaria, but, by extension, it may also explain why medicinal plants with no known effect on disease pathogenesis may relieve users of symptoms associated with the disease and thus improve their quality of life.

Aggravation of symptoms such as weight loss, hypothermia (characterized by reduced/low temperature), and anaemia (characterized by a decreased hematocrit) is parasite load-dependent as mentioned earlier. The weight of mice did not change in the suppressive study but there was weight loss in the curative study and this can be ascribed to the high parasite load. The loss in weight was, however, reversed in treated animals, hence the small positive change in body weight of animals in the curative study. The positive change in weight in the suppressive study could be ascribed to normal growth in the mice as both the infection and the drug showed no effect on the weight of the mice.

The mice experienced hypothermia due to infection. The level of hypothermia is indicated by the negative change in temperature which is higher for the curative study than the suppressive study due to the high parasite load associated with the curative study. The high hematocrit level in the suppressive study could be accounted for by the low parasite load and the related reduced parasite-induced haemolysis associated with the suppressive study. On the other hand, the high parasite load associated with the curative study led to an increased parasite-induced haemolysis resulting in low hematocrit.

The acute toxicity study of the extract recorded an LD_50_ above 2000 mg/kg body weight. In addition, no adverse effects in terms of mortality and behavioural changes either by apathy or reduced locomotion were noticed in the test animals. This is significant since the plant is a usual component of herbal formulations formulated solely or in combination with other plants. The acute toxicity results tell the safety of the plant used in traditional medicine.

*S. cordifolia* powder contained common phytoconstituents including tannins, reducing sugars, saponins, coumarins, and triterpenoids. Ranjani Sivapalan [48] reported the presence of alkaloids, flavonoids, and phytosterols in addition.

## 6. Conclusion

The aqueous extract of *S. cordifolia* has antimalarial properties and is not toxic. It prolongs the survival of infected mice. Bioproperties other than just antiplasmodial properties of the phytoconstituents might be mainly responsible for the extended survival of the extract-treated-infected mice. *Sida cordifolia*, thus, has antimalarial properties and lifespan extension effect in *Plasmodium berghei*-infected mice. This, therefore, rationalizes the traditional use of *S. cordifolia* for the treatment of malaria in Ghana.

## Figures and Tables

**Table 1:** Effect of *S. cordifolia* extract on *P. berghei*-infected mice.

	Suppressive test		Curative test	
Dose mg/kg	% of parasitaemia suppression	Median survival time	% of parasitaemia suppression	Median survival time
NC	0.00	9	0.00	8
50	42.96 ± 0.32^a^	13	37.34 ± 0.25^a^	9.5
100	53.49 ± 0.25^a^	16.5	47.52 ± 0.42^a^	13.5
200	66.13 ± 0.27^a^	19.5	53.80 ± 1.12^a^	17
400	76.90 ± 0.64^a^	26	61.50 ± 0.97^a^	25.5
PC	95.12 ± 0.18^a^	29.5	92.44 ± 0.16^a^	29.5

Values were presented as mean ± SEM; *n* = 4; NC: vehicle-treated group; PC: positive control group (artesunate treated). ^a^Values were significantly different at *p* < 0.0001 when compared with the negative control (NC).

## Data Availability

The data used to support the findings of this study are available from the corresponding author upon request.
